# The Origin and Function of Anti-Fungal Peptides in *C. elegans*: Open Questions

**DOI:** 10.3389/fimmu.2012.00237

**Published:** 2012-08-01

**Authors:** Nathalie Pujol, Paul A. Davis, Jonathan J. Ewbank

**Affiliations:** ^1^Centre d'Immunologie de Marseille-Luminy, UM2 Aix-Marseille UniversitéMarseille, France; ^2^INSERM, U1104Marseille, France; ^3^CNRS, UMR7280Marseille, France; ^4^European Molecular Biology Laboratory-European Bioinformatics InstituteCambridge, UK

While innate immunity has been studied in insects since the time of Pasteur and Metchnikoff (Brey, 1998), research into nematode immune defenses was initiated only comparatively recently (Kurz and Ewbank, 2003). In the mid 1990s, through a biochemical approach, Yusuke Kato was able to isolate an antibacterial activity from the body fluid of the parasitic nematode *Ascaris suum*. This activity was ascribed to *A. suum* antibacterial factor (ASABF), a peptide that is particularly potent against Gram-positive bacteria (Kato, 1995). The subsequent molecular characterization of ASABF allowed the identification of six orthologous “ABF” peptides in the model nematode *C. elegans*, one of which, ABF-2 displays *in vitro* microbicidal activity against a range of bacteria and fungi (Kato and Komatsu, 1996; Kato et al., 2002; Zhang and Kato, 2003). The six ABF peptides were immediately recognized as sharing features with defensins, placing them in the class of cysteine-stabilized α-helix and β-sheet (CSαβ) peptides, the most wide-spread and conserved class of antimicrobial peptides (AMPs; Zhu, 2008). The constitutive expression of *abf-1* and *abf-2*, the best characterized of the six corresponding genes in *C. elegans*, overlaps in the pharynx (Kato et al., 2002); they are likely to contribute to the breakdown of the microbes that form the nematode's normal diet. Although they undoubtedly act upon both bacteria and fungi, here, we consider only their potential role in anti-fungal defenses. Both *abf-1* and *abf-2* are up-regulated by infection with the fungus *Cryptococcus neoformans* (Means et al., 2009), while the expression of *abf-1* but not of *abf-2* is induced by infection with the natural fungal pathogens *Drechmeria coniospora* and *Harposporium* sp. (Engelmann et al., 2011). Conversely, *abf-2* is up-regulated by *Candida albicans*. It is not clear what the underlying regulatory pathways governing *abf* gene expression are (Pukkila-Worley et al., 2011), but one can speculate that their differential regulation reflects both the various modes of pathogen infection (Labed and Pujol, 2011), and also their potentially distinct spectra of antimicrobial activities.

This conserved family of defensin-like peptides is something of an exception, since overall, *C. elegans* possesses a highly derived innate immune system. It has no equivalent of NF-κB, central to immunity in many animals, and also lacks orthologs of most of the receptors known from other species to be important for triggering host defenses (Pujol et al., 2001; Gravato-Nobre and Hodgkin, 2005). Indeed, as explained more in detail below, several classes of AMPs implicated in anti-fungal defense appear to be restricted to certain nematode species, and are controlled by signal transduction cascades with a very limited phylogenetic range (Dierking et al., 2011; Labed et al., 2012). Different fungal pathogens infect either via the intestine following ingestion, or via the epidermis. They influence AMP gene expression via distinct signaling cascades, but a detailed discussion of these regulatory mechanisms is beyond the scope of this short article.

Unlike *A. suum*, which can grow to a length of 40 cm, an adult *C. elegans* measures barely 1 mm, making extraction of body fluid technically almost impossible. Many of the other putative AMP genes in *C. elegans* were initially identified on the basis of their differential regulation following *D. coniospora* infection. The first such genes were members of the *nlp* (for neuropeptide-like protein) and *cnc* (caenacin) families (Couillault et al., 2004). At the time, the former had been tentatively annotated as neuropeptides, based on their limited sequence similarity with known neuropeptides. It was, however, observed that these genes were not generally expressed in the nervous system, but rather in the epidermis (Nathoo et al., 2001). This ties in with the fact that *D. coniospora* spores attach to the nematode's cuticle and then germinate, penetrating into the body of the worm via the epidermis. Much, but not all, of the response to infection is a cell-autonomous mechanism acting in the epidermis; reviewed in Labed and Pujol (2011). The infection-induced *nlp* genes are structurally related to the *cnc* genes. The two groups further share the property of being found in clusters in the genome. Interestingly, comparison of the syntenic regions in two other *Caenorhabditis* species, *C. briggsae* and *C. remanei* revealed that these genes are undergoing relatively rapid evolution, with clear evidence for gene duplication and gene loss, and appear to be under positive selective pressure. Indeed, over-expression of either the *nlp* or *cnc* AMPs leads to somewhat increased resistance to *D. coniospora* infection (Pujol et al., 2008; Zugasti and Ewbank, 2009), suggesting that they play a direct role in host defense against invasive fungi. This is further reinforced by the finding that the *nlp* and *cnc* AMP genes feature prominently among the few genes commonly up-regulated by *D. coniospora* and the fungi *Harposporium* sp. (Engelmann et al., 2011). The expression of *cnc*-*4* and *cnc-7* is also induced by virulent *C. albicans* (Pukkila-Worley et al., 2011), which like *Harposporium* sp. infects *C. elegans* via the intestine.

**Figure 1:**
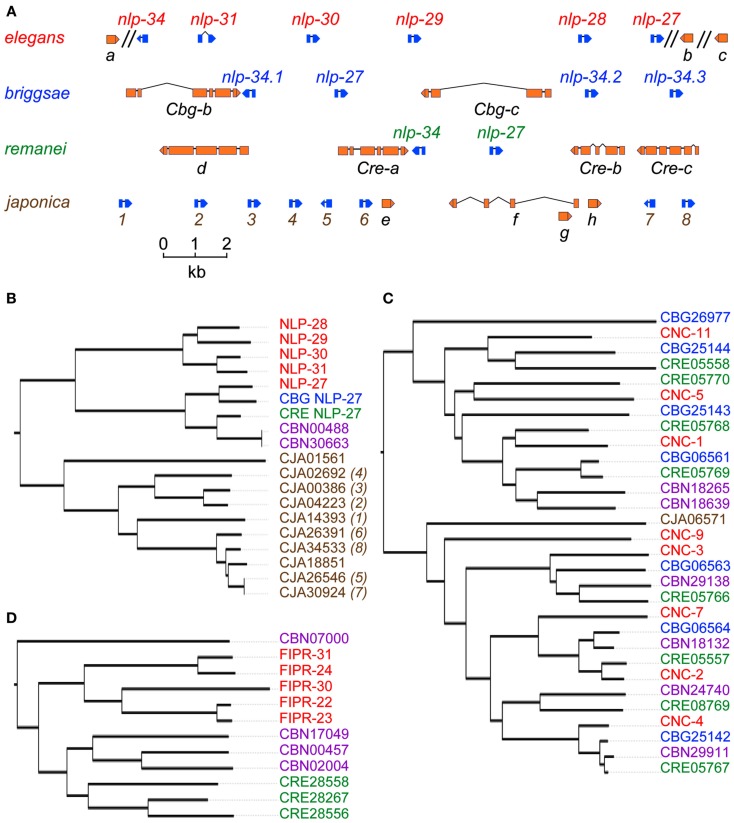
**(A)** The *nlp-29* cluster in *C. elegans* together with syntenic regions from *C. brigssae* (Cbg), *C. remanei* (Cre), and *C. japonica*. AMP genes of the *nlp* class are shown in blue; there has been a marked expansion in *C. japonica*. The genes labeled “*a*” and “*c*” in *C. elegans*, and their orthologs (*Cbg-a*, *Cbg-c*, *Cre-a*, and *Cre-c*) are predicted to encode serpentine receptors; “*b*” in *C. elegans* is K09D9.9. Its ortholog in *C. japonica* is ca. 60 kb 3′ of the locus. Immediately 3′ of the gene labeled “*e*” in *C. japonica* there is the remnant of a degenerate paralog of K09D9.9. The figure is adapted from data in Wormbase WS230; it does not reproduce the predicted fusion of *Cre-b* and *Cre-c*, as this does not appear to be probable. Only the 3′ extremities of *a*, *b*, and *c* are shown. **(B–D)** Phylogenetic trees for selected members of the NLP **(B)**, CNC **(C)**, and FIPR **(D)** family peptides, including homologs in *C. brenneri* (CBN). A distance matrix analysis was performed using the alignment program clustalw2 to generate a guide tree via pairwise and subsequent multiple sequence alignment. This guide tree was then used to produce a true phylogenetic tree that was loaded into the Interactive Tree Of Life v2 software suite (Letunic and Bork, 2011). Partial rooted trees were extracted that corresponding to interesting features within the complete FIP/FIPR/NLP/CNC tree. For the non-*elegans* peptides, with the exception of CBG NLP-27 and CRE NLP-27, the corresponding gene identifier is given. The numbers in brackets for the *C. japonica* gene names match the corresponding genes in **(A)**.

Since the initial phylogenetic studies, more nematode genome sequences have become available. Comparative analyses reinforce the notion of rapid gene evolution. For example, the single copy *C. remanei* gene CRE-*nlp-27* corresponds to a cluster of 5 paralogs in *C. elegans* (Pujol et al., 2008), but 10 predicted paralogs in *C. japonica* (Figures 1A,B). On the other hand, there apparently has been no expansion of the *cnc* genes in *C. japonica*, whereas multiple paralogs are found in the other *Caenorhabditis* species (Figure 1C). We have not found orthologous genes in several other nematode species for which genome sequences are available, including *Brugia malayi* (Ghedin et al., 2007), *Meloidogyne incognita* (Abad et al., 2008), and *A. suum* (Jex et al., 2011). It is possible that these parasitic nematodes have a natural environment that shields them from fungal pathogens throughout their life cycle, so that they have been able to dispense with these AMP genes (Abad et al., 2008). On the other hand, there do appear to be two similar AMP-encoding genes in *Drosophila*, CG7738, now called CG34227, and CG17738 (Couillault et al., 2004). It is notable that the former was found to be up-regulated by fungal infection (De Gregorio et al., 2002), and the corresponding peptide was identified via the biochemical characterization of hemolymph from septically injured flies (Verleyen et al., 2006). The extremely repetitive nature of these peptides, characterized by the presence of multiple GGY and/or GGW triplets (Couillault et al., 2004), does makes identification of orthologs in more distant species problematic, and it is currently unclear how evolutionary ancient are the NLP/CNC AMPs as defense molecules. Nevertheless, the existence of CNC-like peptides in insects supports the idea that the parasitic *A. suum* lost this part of its immune defenses, while retaining defensin-like peptides that may be important in the bacteria-rich intestinal tract of its vertebrate hosts, and also that the primary target of CNCs may be fungi.

The ABFs, NLPs, and CNCs are not the only predicted AMPs in *C. elegans*. Microarray-based transcriptionally profiling of infected worms also led to the identification of seven *fip* (fungus-induced peptide) genes that not only were strongly up-regulated by *D. coniospora*, but that also fulfilled at least three of the four following criteria: (i) predicted to encode a protein of less than 100 amino acids, (ii) with a predicted signal peptide, (iii) judged by inspection to have a simple primary structure (iv) having a homolog in the immediate genomic proximity, or similar in sequence to more than one structurally related protein encoded by clustered genes. Although there is currently no direct evidence to indicate that these genes do encode genuine AMPs, the combination of the above criteria make it highly likely. A larger class of 29 genes, called *fipr* (*fip*-related) was also created for genes that shared these characteristics, but for which there was no indication of any transcriptional up-regulation after *D. coniospora* infection.

This distinction subsequently turned out to be somewhat premature, since an RNAseq-based analysis of the transcriptional response to infection showed that *fipr-22*, *fipr-23*, and *fipr-26* are up-regulated by *D. coniospora*, while the expression of five others (*fipr-1*, *2*, *16*, *17*, and *20*) is induced by the fungus *Harposporium* sp. (Engelmann et al., 2011); *fipr-22* and *fipr-23* are also induced by *C. albicans* (Pukkila-Worley et al., 2011). Because of their large number, and their relatively simple structure, establishing solid phylogenetic relationships both within the nematode clade and beyond is a difficult, and on-going task. It is already clear, however, that these genes too are undergoing relatively rapid evolution, presumably a consequence of their role in host defense. For example, in *C. elegans* there are five paralogous *fipr* genes (including the infection-regulated *fipr-22* and *fipr-23*) clustered together, for which orthologs have not been identified in *C. japonica* or *C. briggsae* (Figure 1D). A very recent study has revealed marked differences in the seasonal abundance of proliferating *C. elegans* and *C. briggsae* populations in Northern France, with *C. elegans* being much more prevalent in the cooler autumn than in summer, when *C. briggsae* predominates (Felix and Duveau, 2012). There is therefore every reason to expect that the two nematode species will not be exposed to exactly the same range of natural pathogens, since microbial communities change with the seasons, perhaps explaining the divergent evolution of their AMP gene repertoire. There is increasing evidence that the commonly used strain of *C. elegans*, N2, underwent a number of changes to adapt to culture under laboratory conditions (see for example, Duveau and Felix, 2012). In the future, it will be interesting to see whether wild isolates of *C. elegans* present variations in their AMP genes.

Most *nlp* genes not regulated by fungal infection encode peptides that are either known to have an endocrine signaling function, or are hypothesized to; certain have been matched to specific G-protein coupled receptors (Husson et al., 2007; Janssen et al., 2010). One cannot exclude the possibility that some of the putative AMPs from the CNC and NLP families also exert a regulatory function during the innate immune response to fungal infection. The metabolism and activity of several classes of neuropeptide, both in *C. elegans* and other species are influenced by neprilysins (Husson et al., 2007). Typically, these zinc metallopeptidases are found on the outer surface of animal cells. They cleave small signaling peptides and thereby block their action. Interestingly, 13 of the 27 neprilysins genes in *C. elegans* are down-regulated upon infection with *D. coniospora* or by *Harposporium* sp. It remains to be determined whether they act on the infection-induced NLPs, or on other classes of peptides, such as insulin-like peptides, which are also transcriptionally regulated upon fungal infection (Engelmann et al., 2011). If they do, this would add a further level of complexity to the regulation of the host response to pathogens.

In conclusion, the last decade has seen considerable advances in our understanding of the role and evolution of AMPs in *C. elegans*. Future studies should yield more insights into their evolutionary origins and conservation, as well as their precise mode of action and the details of their complex regulation.
